# Progress in Medicine

**Published:** 1900-12-22

**Authors:** 


### Progress in Medicine.

### RESPIRATORY DISEASES.

**Phthisis.**—Maffucci[1] maintains that there is no transmitted hereditary predisposition to phthisis, but that the children of phthisical parents may receive the specific germ in fœtal life, or whilst living in intimate contact with their parents. From a series of experiments he concludes that whilst the tubercle bacillus may be conveyed in semen ovum, or placenta, yet more commonly the toxin only is conveyed. The presence of this toxin manifests itself by defective development, abortion, premature labour, cachexia, and increased mortality after birth. Embryonic tissues have greater germicidal powers than adult tissues.

**Diagnosis.**—The value of tuberculin as an aid to diagnosis is urged by McCall Anderson,[2] and details of a series of cases are given. Tuberculin is of especial service in the differentiation of doubtful external lesions, ulcers, scrofuloderma, lupus vulgaris, lupus erythematodes, and adenitis. For diagnostic purposes injections of the old tuberculin are to be preferred, as the reaction is much more pronounced than with the new. The initial dose of the old preparation is from half to one cubic centimètre of 1 in 1,000. This view is also supported by Anders,[3] who maintains that an earlier and more certain diagnosis can be obtained by the injection of tuberculin than by any other method. He gives a very full table showing the names of observers who have tried this method, together with their results. A small proportion of non-tuberculous cases do give a reaction, and a still smaller proportion of undoubtedly tuberculous cases fail to do so; but if the characteristic grouping of features only (an elevation of temperature to 101° F. inclusive) is regarded as a positive reaction, sources of error can be reduced to a minimum. A medium-sized initial dose, from two to five milligrammes, should be employed. The fear that the injections of tuberculin may lead to dissemination of bacilli is now very widely discredited.

Whilst the finding of tubercle bacilli in the sputum is undoubted evidence of the existence of tuberculons disease of the respiratory tract, failure to find them does not entirely negative such a diagnosis,[4] for until the tubercles begin to break down the bacilli are not set free. Thus, in early cases of phthisis, and in cases of miliary tuberculosis, they may be absent. The associated micro-organisms are strepto-, staphylo-, and pneumo-cocci, micrococcus tetragenus, and bacillus pyocyaneus. Strepto-cocci are chiefly responsible for the hectic fever, chills, and night sweats; staphylo-cocci for the suppurating and softening processes. Michaelis[5] finds that the urine of phthisical persons may give the diazo reaction, but only when the disease is advanced or severe, so that it is rather of service for prognosis than diagnosis.

Both at the International Congress of Tuberculosis at Naples[6] and at the American Medical Congress the subject of the earliest symptoms of phthisis was discussed. At the former Bozzalo mentioned the following prodromal signs:—(1) Albuminuria alternating with phosphaturia; (2) anæmia; (3) gastric disturbances; (4) tachycardia and lowered blood-pressure; (5) an excessive rise of temperature following mental or physical exertion, or at the menstrual period; (6) profuse sweating; (7) mydriasis; (8) herpes zoster; (9) enlargement of spleen. Anders, of Philadelphia, advocated the early use of X-rays and the application of the tuberculin test. He found that in early phthisis there was an abnormal rise of temperature between 12 and 2 P.M. Fernet[7] states that in the very earliest stage of the disease there may be found on the side of the affected apex an area of dulness at the base, fine crepitations, and other signs of engorgement. Enlargement of the lymphatic glands in the axilla of the affected side is common, and the bronchial glands are implicated from the first, as is evidenced by the impaired percussion-note in the interscapular region, cavernous breathing, and exaggerated expiratory murmur. This early implication of the bronchial glands was well illustrated in a case reported by Walsham,[8] in which there was in the lung only a single caseous nodule the size of a pea, but a large mass of bronchial glands typically tuberculous in character. The cervical glands, too, were enlarged, and showed large-celled indurative hyperplasia, a condition generally now acknowledged to be a phase of tubercular disease.

**Complications and accompaniments of Phthisis.** The co-existence of mitral stenosis and phthisis has led certain observers—Teissier, Sansom, and others—to conclude that this cardiac lesion has a tuberculous origin. This view is opposed by Walsham,[9] who states that the conjunction is an extremely rare one, and that generally valvular disease with phthisis is rare. In a series of 130 post-mortem examinations on persons dead of pulmonary tubercle, 21 showed evidence of valvular disease, but in only one of these was there stenosis found. Potain,[10] on the other hand, maintains the absolute causal relationship between mitral stenosis and chronic phthisis, especially phthisis of the sclerosing type in arthritic subjects. A pure form of stenosis is produced with no retraction of the cusps, this being the result of a sclerosing endocarditis. There is in these cases a tendency for the pulmonary lesion to become quiescent, and the cardiac condition to be aggravated. Tubercle may also infect the myocardium or endocardium, or may determine an ulcerative endocarditis of secondary coccal origin. Pulmonary stenosis appears to render the lungs susceptible to tubercle. Boardman Reed[11] summarises the gastric changes in phthisis as follows:—In the early stages there is excessive secretion of hydrochloric acid, and thus cod-liver oil, which depresses the stomach functions, is indicated; whilst later, when secretion is inactive, a stimulating remedy, such as creosote, is more beneficial. The motor function, which is generally depressed, is best restored by diet, exercise, fresh air, and abdominal massage. Gant[12] finds that from four to six per cent. of all phthisical persons suffer from fistula, whilst a much larger percentage of those afflicted with fistula have phthisis. True tuberculous fistulæ are secondary to intestinal ulceration; the openings are large and triangular, with the skin edges blue and undermined. The discharge is profuse, whitish, and watery, and contains tubercle bacilli. To obtain a specimen of the discharge, it may be necessary to harden the fæces by the internal administration of laudanum, and then examine the muco-purulent material on the mass. The majority of fistulæ in phthisical persons are non-tuberculous, and are caused by the excessive strain thrown on the lower bowel and perineum by the act of coughing. They are difficult to heal, as the parts cannot be kept at rest, and the patients are prone to suppuration.

Pneumonic complications of phthisis are not uncommon. According to Ophüls,[13] they may be due either to the tubercle bacillus, or to a mixed infection of tubercle bacilli, cocci, and other bacteria. He found pneumonic patches in twenty-five out of thirty-seven successive cases examined. The purely tuberculous pneumonia may be either chronic or acute, and may terminate in caseation and suppuration, or in simple carnification. Generally the caseation and suppuration were hastened by secondary invasion. Of pneumonic complications, due to mixed infection, the acute forms resemble ordinary broncho-pneumonias, but contain epithelioid cells in the exudate; the chronic forms have a tendency to caseate. The pneumococcus is the organism most frequently found in these mixed cases, and it is rare to find the tubercle bacillus absent. As in other prolonged illnesses, the infection is apt to become generalised towards the end, and at the post-mortem organisms may be found in other tissues. Elting[14] gives the following as the main clinical features of acute pneumonic phthisis:—Fever, first of a regular and then of a remittent type. Absence of marked dyspnœa and cyanosis. Consolidation of part or the whole of one lobe; pain in the side; cough, with rusty, glairy sputum, containing tubercle bacilli; absence of leucocytosis. Philip[15] concludes that pneumonia is very rarely found in immediately determinate relationship with pulmonary tuberculosis, and that most of the cases recorded may be explained by the pre-existence of undetected tuberculous foci, or by delayed resolution. The diagnosis must rest on the finding of the tubercle bacillus.

[1] Rev. Crit. de Clin. Med., March 24, 1900. [2] Lancet, June 16, 1900. [3] New York Med. Jour., June 23, 1900. [4] Med. Rec., July 14, 1900. [5] Berl. Klin. Woch., Nov. 13, 1900. [6] Lancet, Aug. 4, 1900. [7] Jour. de Bact., Aug. 5, 1900. [8] Brit. Med. Jour., Nov. 18, 1899. [9] Ibid., Oct. 28, 1899. [10] Jour. de Med., April 10, 1900. [11] Ep. 449, Brit. Med. Jour., June 16, 1900. [12] Med. Rec., July 7, 1900. [13] Amer. Jour. Med. Sci., July, 1900. [14] Ibid., May, 1900. [15] Practitioner, Feb. 1900.

